# Cautious addition of targeted therapy to PD-1 inhibitors after initial progression of BRAF mutant metastatic melanoma on checkpoint inhibitor therapy

**DOI:** 10.1186/s12885-021-08906-1

**Published:** 2021-11-07

**Authors:** Wolfram Samlowski, Camille Adajar

**Affiliations:** 1 Comprehensive Cancer Centers of Nevada, 9280 W. Sunset Rd., Suite 100, Las Vegas, NV 89148 USA; 2 University of Nevada Las Vegas, (UNLV) Kerkorian School of Medicine, Las Vegas, NV USA; 3 University of Nevada School of Medicine, Reno, NV USA

**Keywords:** Checkpoint inhibitor, Ipilimumab, Nivolumab, Pembrolizumab, BRAF inhibitor, MEK inhibitor, Immunotherapy

## Abstract

**Background:**

Virtually all metastatic patients with metastatic melanoma who progress after initial treatment with PD-1 or CTLA-4 directed antibodies will die of their disease. Salvage options are urgently needed. It is theoretically attractive to combine immunotherapy with targeted agents in progressing patients with BRAF mutation positive melanoma, but the toxicity of combined treatment has proven challenging.

**Methods:**

We performed a retrospective analysis of our patient database and identified 23 patients who progressed on initial checkpoint inhibitor treatment, who subsequently had cautious addition of BRAF±MEK inhibitor therapy to continued PD-1 antibody treatment.

**Results:**

We found an objective response rate of 55% in second line therapy, with a median progression-free survival of 33.4 months and median overall survival of 34.1 months, with 40% of patients in unmaintained remission at over 3 years. Ten of 12 responding patients were able to discont**i**nue all therapy and continue in unmaintained remission. Toxicity of this approach was generally manageable (21.7% grade 3–5 toxicity). There was 1 early sudden death for unknown reasons in a responding patient.

**Discussion:**

Our results suggest that 2nd line therapy with PD-1 inhibitors plus BRAF±MEK inhibitors has substantial activity and manageable toxicity. This treatment can induce additional durable complete responses in patients who have progressed on initial immunotherapy. These results suggest further evaluation be performed of sequential PD-1 antibody treatment with cautious addition of targeted therapy in appropriate patients.

## Background

Melanoma represents a significant public health issue in the United States. The incidence of melanoma has been increasing over many decades [1]. In 2020, it is estimated that there were 100,350 patients diagnosed with invasive melanoma, resulting in 6850 deaths [1]. During the last 2 decades, dramatic changes in treatment of metastatic melanoma and improvements in patient survival have taken place. These have originated from two conceptual advances: 1) There has been recognition that there is a pattern of recurrent somatic genetic mutations in cancers that drive melanoma growth and metastases [2]. This has led to development of “targeted” therapy (TT) agents [3]. 2) It has been recognized that cancers are not immunologically silent and that cancer progression requires that cancers evade the immune system detection and destruction [4]. This has led to the discovery of immune checkpoints that activate or inhibit T cell responses to cancer. The impact of these discoveries has been that in 2005, the median survival of a patient with metastatic melanoma treated with chemotherapy was only 6–7 months, with only 25% alive at 1 year [5]. In 2019, the median survival of metastatic melanoma had increased to over 50% alive at 5 years, a statistic that hopefully will continue to improve with further treatment advances [6, 7].

TT in melanoma has generally been directed at the most common activating oncogene mutation. This mutation occurs in the tyrosine kinase BRAF and is most commonly a single nucleotide substitution at V600E [3]. BRAF V600E mutations appear to be present in approximately 40–50% of skin melanomas [8]. It has become apparent that simultaneous blockade of the mutated BRAF and additional members of the downstream signaling pathway (vertical blockade) such as the oncogene MEK, using combinations of BRAF+MEK inhibitors increases the effectiveness of TT while maintaining tolerable levels of side effects [9].

BRAF ± MEK inhibition has been shown to induce very rapid responses in the majority of metastatic BRAF-mutant melanomas [10]. Eventually most of the responding patients develop adaptive resistance to TT over a median span of 12–18 months [11]. With 5-year follow-up, only 19% of patients treated with dabrafenib (BRAF V600E inhibitor) plus trametinib (a MEK inhibitor) continue to have ongoing responses of their melanoma despite continual therapy [7].

Recognition that cancers must evade the immune system to progress has led to additional cancer treatment advances. Responses to specific tumor antigens requires not only the antigen recognition site on T cells (the T cell receptor), but also engagement of co-stimulatory and co-inhibitory receptors. These additional receptors modulate immune responsiveness (termed immune checkpoints) [12, 13]. Cancer cells appear to hijack the inhibitory pathways to prevent recognition and destruction by the immune system [14]. Thus, checkpoint inhibitor (CKI) antibodies blocking the function of co-inhibitory T cell receptors (e.g., CTLA-4 and PD-1) have proven powerful in reviving “exhausted” immune responses in patients with melanoma and other cancers. These agents re-activate anti-melanoma immunity against cancers [15]. In metastatic melanoma, combined PD-1 + CTLA-4 inhibition has produced 5-year progression free survival rates of 37% with 5-year overall survival exceeding 50%. Monotherapy with PD-1 antibodies or CTLA-4 antibodies individually have achieved somewhat lower rates of durable complete remissions in patients with melanoma [6, 16].

It seemed reasonable, therefore, to investigate whether a combination of TT and CKI is feasible [17]. This would theoretically take advantage of the rapid onset and depth of response achievable with targeted agents with the durability of response and long-term survival benefit seen with immunotherapy. There is developing clinical evidence that BRAF±MEK inhibitor therapy also results in significant immunologic potentiation [18]. In addition, targeted agents may increase immune infiltration into tumors and enhance T cell cytotoxicity [19], in part via increased PD-1 ligand expression on melanoma cells [20]. TT may also increase melanoma specific antigen expression, enhance dendritic cell function, increase NK and T cell function, and overcome inhibitor influences in the tumor microenvironment [17]. Unfortunately, combinations of CKI and TT have proven challenging, due to an apparent significant increase in toxicity related to the combination of TT with concurrent CKI, even when these agents are given sequentially without an adequate washout period [21, 22]. Thus, clinical trials of TT in combination with CKI are still in their infancy.

We have treated a sizeable cohort of clinical trial ineligible patients with CKI antibodies. In patients progressing following CKI treatment, the vast majority of whom would eventually die from metastatic melanoma, we have cautiously added low doses of targeted agents when a druggable mutation was present (e.g., BRAF with or without MEK inhibitors) with the hypothesis that these agents would slow cancer growth to provide time for productive immune responses to develop and perhaps to take advantage of mmune potentiation induced by BRAF±MEK inhibitors [23–26].

We have observed significant responses including conversion of rapidly progressing patients to durable complete responses with acceptable levels of toxicity in BRAF mutant melanoma with the addition of low doses of TT in patients whose metastatic tumors progressed after treatment with CKI antibodies. In this retrospective analysis, we have analyzed and quantified the clinical activity and side-effects of CKI + TT in metastatic patients with BRAF mutant metastatic melanoma who progressed on their initial immunotherapy.

## Methods

### Study design

A retrospective chart review was conducted of patients with BRAF-mutant metastatic melanoma treated by a single physician (WS) at the Comprehensive Cancer Centers of Nevada between 2014 and 2020. Mutation status was evaluated by next-gen sequencing (Genoptics, Carlsbad CA or Foundation Medicine, Cambridge MA). A secure HIPAA compliant iKnowMed data base (McKesson, Houston, TX) was searched for patients with BRAF mutant metastatic melanoma who had received treatment with BRAF inhibitors (vemurafenib, dabrafenib, encorafenib) with or without MEK inhibitors (cobimetinib, trametinib or binimetinib). Charts from these patients were reviewed to identify patients who had initially been treated with checkpoint inhibitor antibodies (pembrolizumab, nivolumab or ipilimumab). Patients who progressed on initial checkpoint inhibitor (CKI) therapy by clinical exam or radiographs were screened. All patients were offered clinical trial participation, when available. Those who were given a targeted therapy (TT) regimen of low dose BRAF +/− MEK inhibitors in combination with continuation of ongoing anti-PD-1 monoclonal antibody treatment were identified and data extracted into a de-identified patient data spreadsheet for analysis (Excel, Microsoft, Redmond WA). This retrospective chart review study was reviewed by the Western Institutional Review Board (IRB) and deemed exempt from full IRB review.

### Evaluation of outcomes

Patient response to initial CKI therapy, was measured from the start of treatment until the start of TT (termed R1) and respective medication doses were recorded. The duration of subsequent combined treatment with CKI + BRAF +/− MEK inhibitors (R2), measured from the start of TT until the date of progression or the last clinic visit (if in remission) was also recorded, as was any possible toxicity. Objective response rate was determined via sequential CT scans using RECIST 1.1 criteria^7^ and overall survival of patients was calculated from the start of CKI treatment. Complete Response (CR) was defined as disappearance of all target and non-target lesions and normalization of tumor marker levels. Partial response (PR) was defined as more than a 30% reduction in sum of bidimensional tumor measurements. Progressive disease was described as > 20% increase in sum of bidimensional tumor measurements or the development of new metastases. Stable disease (SD) was defined as any response not meeting criteria for CR, PR or PD. Data collection concluded December 31, 2020.

### Treatment regimens

In our practice, patients with BRAF-mutant melanomas were virtually always treated initially with a CKI regimen, such as pembrolizumab, nivolumab, or the combination of ipilimumab plus nivolumab due to frequent delays in obtaining BRAF sequencing data. If the melanoma progressed on CKI therapy, patients with a BRAF mutation were offered cautious addition of a low dose BRAF +/− MEK inhibitor with continuation of PD-1 antibody therapy. TT typically consisted of dabrafenib 75 mg/day with or without trametinib 1 mg/day or encorafenib 75 mg/d with or without binimetinib 15 mg b.i.d. If there was no apparent toxicity after a week of concurrent therapy, cautious dose escalation of BRAF or MEK inhibitors was considered. Patients who were given TT before CKI or who were given CKI and TT simultaneously as initial therapy were excluded from the analysis. The final cohort included 23 patients who met the described study parameters. Patients were closely monitored for signs of toxicity, and treatment was interrupted if toxicity persisted or could not be controlled with dose-reduction or adjunctive treatment.

### Statistical analyses

Descriptive statistics were calculated via Excel spreadsheet. Survival was calculated from the start of CKI therapy until the last visit if the patient was still alive or the date of death if the patient was deceased. Best response was determined based on if the patient had a complete response, partial response, stable disease, or progressed a second time after the combined therapy. The data analysis cutoff date was 12/31/20. A Kaplan Meier analysis was performed to evaluate progression-free and overall survival [27].

## Results

### Patient characteristics

The screened population consisted of 53 patients with BRAF-mutant melanoma who were treated with initial CKI treatment. It should be noted that approximately 65% of our BRAF-mutant patients progressed after initial immunotherapy. The eligible study population consisted of 23 patients. Patient demographics and disease characteristics are presented (Table 1). In this cohort of patients, 18 (78.3%) had a V600E mutation, 3 (13.0%) had a V600K mutation, and 2 patients (8.7%) had other BRAF mutations (a BRAF T599_V600 ins T, and a c1794–1796 duplication). In addition, two (8.7%) patients had concomitant loss of both CDKN2A/B and 1 (4.3%) had a CDK/N2A exon 2 deletion. Six patients (26.1%) had a concomitant TERT promoter mutation.

**Table 1:** Patient characteristics at time of initial immunotherapy

Pt	Sex	Age range	Site	Mutation	Initial LDH	Metastatic sites	TMB (per Mb)	PDL1	Brain mets	KPS (%)	Initial CKI	R1 (mo)	CKI toxicity
1	F	50–60	Leg	V600E	nl	2	NA	NA	+	90	P	4.6	none
2	F	40–50	back	V600E	nl	1	3	3%	–	100	N	6.8	none
3	M	20–30	back	V600E	nl	> 3	5	NA	–	100	I/N	5.1	fevers, chills, sweats, diarrhea
4	M	60–70	scalp	V600E	nl	> 3	NA	1%	–	90	P	2.8	headaches, syncope
5	M	30–40	finger	V600E	nl	2	6	NA	–	100	N	10.3	diarrhea
6	F	20–30	neck	V600E	↑	> 3	NA	NA	+	90	I	1.1	none
7	F	50–60	arm	V600E	nl	1	10	NA	–	100	N	2.8	rash
8	M	40–50	ear	V600K	nl	2	25	NA	–	90	I/N	4.6	none
9	F	40–50	arm	V600E	nl	> 3	NA	NA	–	100	I/N	0.7	none
10	F	50–60	leg	V600E	nl	> 3	NA	NA	–	90	I/N	0.5	none
11	M	20–30	leg	V600K	nl	> 3	NA	NA	–	100	N	2.3	none
12	M	40–50	leg	V600E	nl	> 3	NA	> 1%	–	90	I/N	4.9	diarrhea, hypopituitarianism, eye pain
13	M	50–60	leg	V600E	nl	> 3	NA	0%	–	100	I/N	0.3	none
14	F	50–60	leg	V600E	↑	2	5	NA	–	100	N	14.7	none
15	M	50–60	back	V600E	↑	> 3	8	NA	+	80	I/N	2.8	none
16	F	60–70	back	V600K	nl	1	929	0%	–	90	I/N	4.0	Headaches, fatigue, hypotension, arthralgias, diarrhea
17	M	80–90	back	V600E	nl	> 3	NA	40%	–	80	I/N	0.7	none
18	M	40–50	leg	V600K	nl	1	NA	NA	–	90	P	3.3	scalp infection
19	M	60–70	back	c1794–1796 dup	↑	> 3	NA	NA	+	80	I	0.3	none
20	F	50–60	back	V600E	nl	3	NA	NA	–	100	P	3.5	none
21	F	50–60	chest	V600E	↑	> 3	NA	NA	+	90	I/N	1.4	none
22	M	50–60	leg	T599-V600 ins T	nl	1	1	NA	–	90	I/N	3.5	none
23	M	30–40	leg	V600E	nl	> 3	NA	NA	–	90	I/N	0.5	diarrhea, nausea, vomiting, abdominal pain, poor appetite

*M* male, *F* female, *nl* normal, *↑* elevated above institution upper limit of normal, *NA* not available; *−* absent, *+* present; Initial checkpoint inhibitor therapy CKI, *I* ipilimumab, *N* nivolumab, *P* pembrolizumab

The median age at diagnosis of metastatic melanoma was 50 ± 14 years (±SD), and the age range was 20–82 years. Thirteen (56.2%) of evaluable patients were male and 10 (43.5%) were female. All 23 patients were Caucasian. Twenty patients have ended treatment, two continue on TT treatment alone in ongoing remission, and one had progressive disease on CKI + TT treatment and is now enrolled on a clinical trial.

The extent of melanoma at the start of TT + CKI therapy is shown in relation to eventual response (Table 2). It is interesting that bulky tumor sites or a miliary pattern of metastases (dozens to hundreds of small metastases at multiple sites) did not prevent the induction of durable complete responses. There appeared to be more patients who developed brain metastases at the time of CKI progression in the group that died (6/10 patients), compared to the responding group (2/13). There also appeared to be modestly more patients with an elevated lactate dehydrogenase (LDH) at progression (5/10) who died compared to the responding group (3/13).

**Table 2:** Melanoma characteristics from start of TT + CKI treatment

Pt	Metastases	additional characteristics	Brain metastases	LDH	PD-1	TT	TT Toxicity	CTCAE grade	R2 (mo)	Outcome
1	SC, LN		+	↑	P	D	nausea, anemia, interstitial nephritis	G4	20.3	CR-Off
2	SC		–	nl	N	DT	chronic nausea	G1	11.2	CR-Off
3	SC, LN, adrenal, liver, spleen, lung, bone	bulky LN disease	–	nl	N	E	hand/foot syndrome, fatigue, floaters	G2	22.6	CR-Off
4	SC, LN,lung		–	nl	P	DT/EB	diarrhea, rash	G2	44.2	CR-Off
5	SC bone		–	nl	N	EB	diarrhea	G2	7.4	CR-TT
6	SC, LN, liver, lung, bone	miliary metastases	+	↑	N	D	none-sudden death	G5	0.4	CR-sudden death
7	SC, LN	bulky LN disease	–	nl	N	DT/EB	rash, keratoacanthomas	G1	22.9	CR-TT
8	SC, LN	bulky LN disease	–	nl	N	DT	seronegative arthritis fatigue	G3	35.5	CR-Off
9	SC, LN, lung bone	miliary metastases	–	nl	N	D	hand foot syndrome, keratoacanthomas	G1	33.4	DOD
10	SC, LN	bulky LN disease	–	↑	N	D	visual blurring, photophobia, DVT, seronegative arthritis	G3	50.6	CR-Off
11	SC, LN, peritoneum, bone	miliary metastases	–	nl	N	D	none	G0	64.6	CR-Off
12	SC, LN, lung bone	miliary metastases	–	nl	N	D	pulmonary granulomas	G2	51.9	CR-Off
13	SC, LN	bulky SC disease	–	nl	N	DT	diarrhea	G2	33.2	CR-Off
14	SC, LN, peritoneal		–	nl	N	EB	nausea, weight loss, abdominal discomfort, shooting pains	G2	18.8	PD-alive
15	SC, intramuscular, LN, peritoneum, spleen, bone	miliary metastases	+	↑	N	EB/ET	None	G0	6.3	DOD
16	SC, LN		–	↑	N	EB/DT	rash, fever, arthralgias, fatigue	G1	9.0	DOD
17	SC, LN		–	nl	N	DT/VC	joint aches, maculopapular rash, pruritis	G2	8.5	DOD
18	SC, LN, bone		+	nl	P	DT	arthralgia	G1	6.3	DOD
19	LN, adrenal, liver, spleen, lung, bone		+	↑	N	DT	none	G0	0.6	DOD
20	lung		–	nl	P	DT	none	G0	15.4	DOD
21	SC, intramuscular, LN, lung, liver, spleen, adrenal		+	↑	N	DT	hypotension, anemia	G1	7.7	DOD
22	lung, liver, LN, bone		+	↑	N	DT/EB	ataxia, sensory motor neuropathy, immune nephritis	G4	9.7	DOD
23	lung		+	nl	N	D	none	G0	2.1	DOD

*SC* subcutaneous, *LN* lymph nodes, *+* present, − absent, *↑* elevated, *nl* normal; Immunotherapy: *N* nivolumab, *P* pembrolizumab, *TT* Targeted Therapy, *D* dabrafenib, *T* trametinib, *E* encorafenib, *B* binimetinib, *V* vemurafenib, *C* cobimetinib, *CR-Off* ongoing complete response, off all therapy, *CR-TT* complete response continuing targeted therapy, *DOD* dead of disease, *PD-alive* progressive disease, remains alive

### CKI treatment characteristics

Initial checkpoint inhibitor therapy consisted of ipilimumab plus nivolumab in 12 patients, pembrolizumab monotherapy in 5 patients, nivolumab monotherapy in 5 patients and ipilimumab therapy (prior to approval of PD-1 antibodies) in 1 patient who subsequently received treatment with PD-1 MAb with added TT. These choices were determined by the timing of regulatory approval of these agents.

### TT treatment characteristics

Initial TT consisted of dabrafenib/trametinib in 11 patients, dabrafenib monotherapy in 7 patients, encorafenib/binimetinib in 4 patients, and encorafenib monotherapy 1 patient. A total of 6 patients required a switch to alternate BRAF/MEK inhibitors due to drug specific toxicity. None of these patients needed to completely discontinue BRAF±MEK directed treatment due to persistent toxicity. Patients treated with dabrafenib monotherapy were treated prior to regulatory approval of the dabrafenib/trametinib regimen.

### Response and survival analysis

Following treatment with combined TT and CKI therapy, a complete response was observed in 13 (56.5%) patients. One patient was found to have a partial response (4.3%), 1 patient had stable disease (4.3%), and 1 patient had an unknown response (4.3%) to combined therapy due to sudden death shortly after the start of the combined regimen. Progressive disease was observed in 8 (34.8%) of patients. Of the 14 complete response patients, 13 (92.9%) are currently alive.

**Fig. 1:**
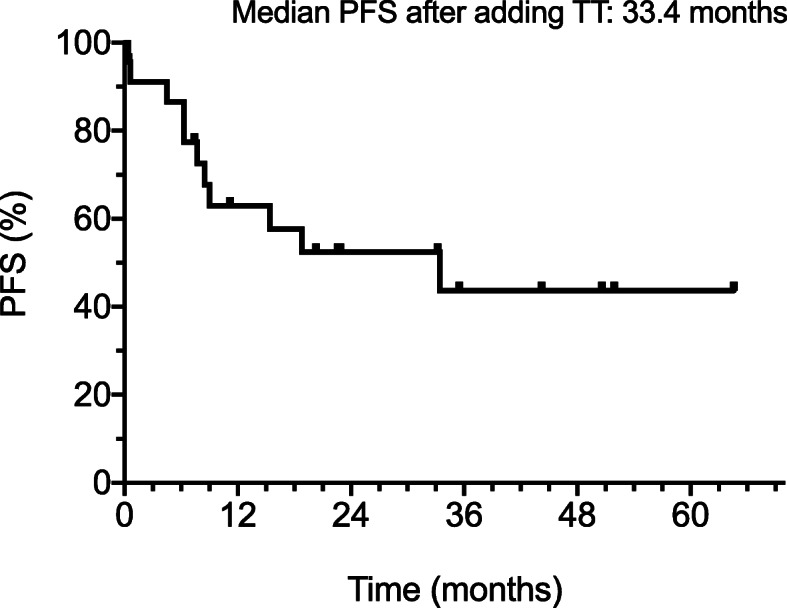
Progression free survival after adding TT to CKI therapy in 23

With over 30 months median follow up, median progression free survival (PFS) from the start CKI treatment and subsequent addition of TT was 33.4 months (R2). The confidence interval for the PFS rate ranged from 35 to 80% at 30 months (Fig. 1). A total of 40% of patients were still progression free beyond 3 years of follow-up.

**Fig. 2:**
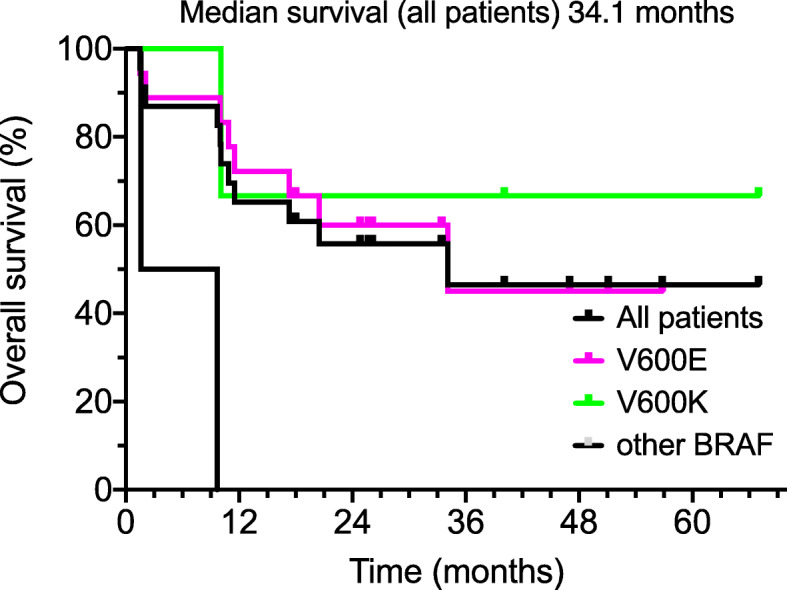
Kaplan-Meier analysis of overall survival of 23 patients treated with TT and CKI therapy

The median overall survival for all patients with BRAF-positive melanoma for patients who received combined 2nd line TT and CKI therapy was 34.1 months from the onset of CKI treatment (Fig. 2). The 95% confidence interval for response probability at 30 months was 35–75%. Patients with a V600E or a V600K BRAF mutation had more frequent durable responses to combined therapy than patients with a BRAF insertion or duplication, who had a very poor survival.

**Fig. 3:**
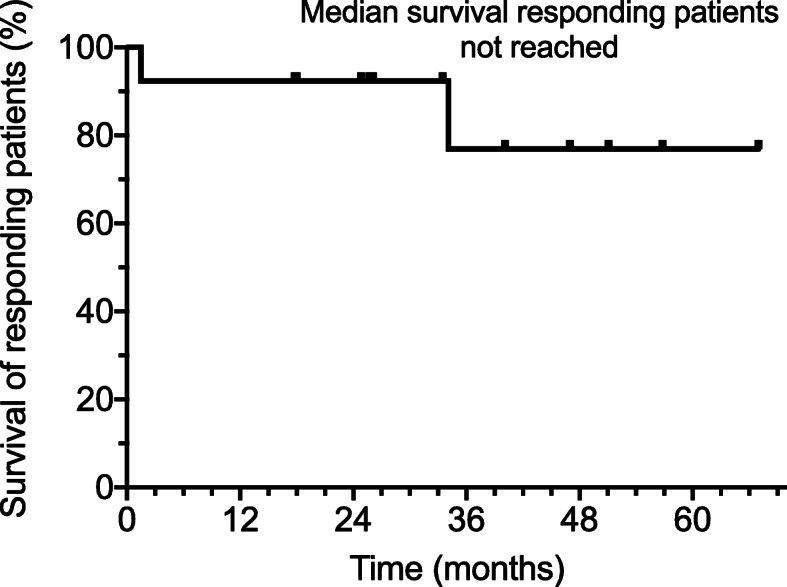
Survival in 12 patients responding after combined TT and CKI therapy

Of the responding patients, median progression free survival of responding patients has not been reached. A total of 11/12 patients continue in an ongoing response and continue to survive (91.7%)(Fig. 3). At over 3 years median follow-up, 76.9% of responding patients remain alive.

### Progression

**Fig. 4:**
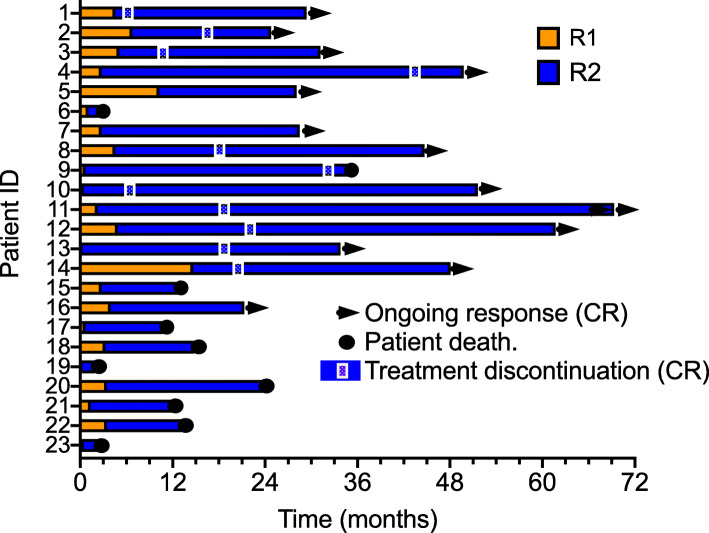
Clinical response to CKI therapy (R1) and subsequent response to CKI + TT treatment (R2)

**Fig. 5:**
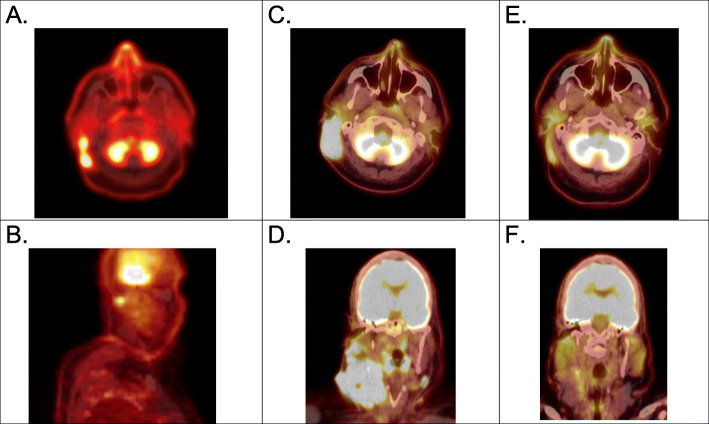
PET/CT scans showing the clinical response of patient 8: Initial pretreatment scan showing melanoma metastatic to (**A**) retro-auricular scalp soft tissues and (**B**) a small ipsilateral neck node. In (**C** and **D**) similar cuts show dramatic worsening of postauricular disease and bulky neck lymph nodes after 4 cycles of ipilimumab plus nivolumab therapy. Clinical response to combination CKI + TT is shown in scalp lesion **E**), as well as the neck adenopathy (**F**) at 40 months from start of CKI. Due to residual low-level fluorodeoxyglucose uptake at both neck and post-auricular sites, both were biopsied, and a pathologic complete response was documented. The patient remains stable at over 9 months after discontinuation of all treatment

Response 1 (R1) was defined from the start of CKI therapy to the start of combined CKI therapy and TT and was calculated for all patients. The median time to CKI progression (P1) was 3.6 months ±4.4 months SD, with a range of 4.6–15.9 months (Fig. 4). Some investigators have termed patients with clinical or radiographic doubling of disease by 2–3 months “hyperprogressors” [28]. These patients are known to have a very poor prognosis. Of the 23 patients in this study 10/23 (43.5%) would fit this definition, including 5 of 14 complete responders. An example (patient 8) is shown (Fig. 5).

Response 2 (R2) was calculated from the start of TT administration until disease progression or the most recent clinic visit if the patient was still alive. The median R2 was 17.1 months ±17.0 months SD (range 0.4–62.6 months). This was longer than the initial CKI response (R1) in every single patient. It should be noted that 10 of 12 responding patients have completely discontinued anticancer therapy using criteria published by Robert et al. [29]. These criteria included 2 negative scans at least 13 months apart, after at least 6 months of therapy. In our patient series10/12 (83.3%) remain in ongoing complete response after treatment discontinuation (Fig. 4). Two patients (16.6%) remain on treatment after complete response for 12 months, one patient was in CR for 33 months, but unfortunately died 3 months after discontinuing all therapy of a catastrophic brain relapse with CNS hemorrhage (8.3%). Active follow-up of responding patients after combined TT and CKI therapy is continuing. In non-responding patients a major cause of mortality was the development of brain metastases in 7 out of 10 patients. The other 3 patients died of systemic metastatic disease progression.

### Toxicity

Toxicity was closely monitored in all patients. If toxicity appeared immune-mediated, immunotherapy was held, and steroid treatment was started. Secondary immunosuppressive agents were used as indicated. Endocrine function was closely monitored, and thyroid or steroid replacements were started when indicated, without treatment interruption. Toxicity from targeted agents was treated as recommended by the manufacturer. Treatment was discontinued if toxicity persisted or could not be adequately controlled with adjunctive measures or reached grade 3 or 4 severity. Significant adverse effects were noted in 10 (43.5%) of patients while on initial CKI therapy. After being started on combined TT and CKI therapy, adverse effects were noted in 19 (79.17%) of patients **(**Table 1). Most treatment toxicity was grade 0–2 and able to be controlled with adjunctive measures, allowing treatment to be continued. The spectrum of observed toxicities seen with both initial CKI treatment (Table 1**)** and subsequent TT plus PD-1 therapy **(**Table 2) is shown. A total of 2 patients (8.7%) had grade 3 seronegative arthritis (treated as an outpatient) and there were 2 grade 4 toxicities that required hospital admission (8.7%). One patient with prior brain metastases died suddenly of uncertain causes (previous EKGs did not show QT prolongation), while dramatically responding to therapy. Unfortunately, no autopsy was permitted. The total incidence of grade III-V toxicities was 21.7%.

## Discussion

Development of CKI and TT have dramatically changed the treatment options for patients with metastatic melanoma. Regardless of treatment choice, eventual disease progression in a majority of patients remains a clinical challenge. For example, patients whose best response to ipilimumab and nivolumab was progressive or stable disease had a median survival of only 3 and 6 months respectively [Long et al., Characteristics of Long-Term Survivors and Subgroup Analyses with Combination Nivolumab Plus Ipilimumab for Advanced Melanoma (CheckMate 067), presented at Society for Melanoma Research 2019]. In a retrospective reanalysis of 3 clinical trials, patients treated second line TT after initial CKI failure had a PFS of only 18% when treated with 2nd line BRAF±MEK inhibitors [30]. Thus, better treatment options for patients with progression of melanoma after initial CKI therapy are still badly needed.

Since targeted agents induce a rapid and deep response with eventual development of adaptive resistance and checkpoint inhibitors produce more gradual onset of activity with a significant fraction of complete and durable responses, combining CKI and TT is highly attractive in BRAF mutant patients. Unfortunately, early attempts at combination therapy led to unacceptably high levels of toxicity [21, 31, 32].

The published toxicity of combination therapy appeared, in part, due to the unanticipated immunologic activity of BRAF and MEK inhibitors. BRAF±MEK inhibitor therapy appears to result in significant immunologic potentiation, by increasing immune infiltration into tumors and enhancing T cell cytotoxicity [18, 19], in part via increased PD-1 ligand expression on melanoma cells [20]. In addition TT may increase melanoma specific antigen expression, enhance dendritic cell function, increase NK and T cell function, and overcome inhibitor influences in the tumor microenvironment [17]. More recently, a number of front-line combination trials of BRAF+MEK inhibitors have reported results, most of which have not met their primary endpoint goals. However, these reports have provided tantalizing hints of potential effectiveness of these regimens [32–34].

In the Keynote 022 trial, patients were randomly assigned 1:1 to receive dabrafenib (150 mg orally two times per day) and trametinib (2 mg orally one time a day) with intravenous pembrolizumab (200 mg every 3 weeks) or placebo. The primary endpoint was PFS. With 36.6 months of follow-up, median PFS was 16.9 months (95% CI 11.3 to 27.9) with triplet and 10.7 months (95% CI 7.2 to 16.8) with doublet (HR 0.53; 95% CI 0.34 to 0.83)(*p* = 0.53). With triplet and doublet, respectively, PFS at 24 months was 41.0 and 16.3%; median response duration were 25.1 months and 12.1 months, respectively. Median OS was not reached with triplet and was 26.3 months with doublet (HR 0.64; 95% CI 0.38 to 1.06). Grade 3–5 treatment-related adverse events (TRAEs) occurred in 35 patients (58%, including one death) receiving triplet and 15 patients (25%) receiving doublet [34].

The IMspire150 trial was a randomized, double-blind, placebo-controlled phase 3 study that enrolled 514 patients. Patients with unresectable stage IIIc–IV, BRAF V600E mutant melanoma were randomly assigned 1:1 to 28-day cycles of vemurafenib (960 mg b.i.d.), and cobimetinib (60 mg/d for 21 days) with either atezolizumab (840 mg) or placebo. At a median follow-up of 18.9 months, progression-free survival as assessed by the study investigator was significantly prolonged with atezolizumab versus control (15.1 vs 10.6 months; hazard ratio [HR] 0.78; 95% CI 0.63–0.97; *p* = 0·025). Treatment-related adverse events occurred in 99% of both the atezolizumab and control groups, with 13% of patients in the atezolizumab group and 16% in the control group stopping all treatment because of adverse events [33].

In the COMBI-I trial, 532 patients were randomized to receive spartalizumab 400 mg or placebo IV every 4 weeks with dabrafenib 150 mg twice daily and trametinib 2 mg daily. At median follow up 27.2 months, the spartalizumab-based regimen did not significantly improve PFS (median 16.2 vs 12.0 months; *p* = .042). Estimated 12- and 24-month PFS rates with the spartalizumab regimen versus placebo were 58% vs 50 and 44% vs 36%, respectively. The objective response rate was 69% in the spartalizumab arm (CR rate 20%) vs 64% in the placebo arm (CR rate, 18%); median duration of response was not reached vs 20.7 months, respectively. Treatment-related adverse events (TRAEs) grade ≥ 3 occurred in 55% vs 33% of patients treated with spartalizumb arm versus placebo. TRAEs leading to discontinuation of all 3 study drugs occurred in 12% vs 8% of patients in the two arms, respectively [35].

We approached the problem of immunotherapy resistant patients somewhat differently than most current trials. In a community setting, there are frequently significant delays in identifying BRAF mutant patients, as reflex BRAF mutation testing is not routinely being performed by community pathologists. Also, V600E-specific mutation testing by monoclonal antibody staining is known to miss a percentage of patients with other BRAF gene mutations (including V600K, V600R and internal gene rearrangements or gene fusions) as well as other potentially targetable mutations, which are more accurately pinpointed via next-gen sequencing panels [36]. Thus, initiating treatment with CKI avoids lengthy treatment delays while awaiting molecular test results. In addition, there is developing data in BRAF mutant patients that initial CKI therapy followed by BRAF inhibition at relapse may have a higher progression free survival than the reverse sequence [30, 37–39].

We also recognized that a substantial percentage of patients (30–40%) treated with initial immunotherapy would achieve complete and durable remissions without any additional therapy. Inclusion of patients with potential CKI response may have confounded analysis of the randomized trials described above and exposed these patients to unnecessary risks for toxicity. BRAF mutant patients may have a modestly increased response potential to CKI treatment compared to non-BRAF mutated patients [6]. It is also known that the majority of patients with best response of progressive or stable disease progression after initial CKI treatment will rapidly die of their disease, with a median survival of 3–6 months [Long et al., Characteristics of Long-Term Survivors and Subgroup Analyses with Combination Nivolumab Plus Ipilimumab for Advanced Melanoma (CheckMate 067), Society for Melanoma Research 2019]. Thus, we chose to treat only patients who had clearly progressed on immunotherapy with cautious addition of TT to ongoing PD-1 directed treatment. This avoided overtreatment of patients who would achieve complete response without addition of TT, avoiding unnecessary additive toxicity of combining CKI and TT. We also discarded the chemotherapy concept of “maximum tolerated dose” unlike the trials cited above, as it is not clear that this applies to either CKI or TT therapy [40–42], instead seeking a minimum effective dose of TT with CKI. Our belief was that this might arrest tumor progression while enhancing potentially synergistic immune activation and decrease the risk of additive toxicity.

Our results support the clinical activity of a sequential treatment approach. We were able to successfully combine CKI with cautious escalation of TT. Our approach was able to produce a significant frequency of complete responses in 55% of patients including a significant number of patients with “hyperprogression”. This included patients with either a BRAF V600E and V600K mutation. Progression free survival at a median follow up at 33 months was 34.1 months. Median overall survival was appeared to show a median survival of 34.1 months with a plateau of 48% after 3 years. Of patients achieving a complete response, 10/13 remain in long term remission. Many of these patients have been able to successfully discontinue all treatment based on criteria published by Robert et al. [29], and remain in ongoing remission. One patient died of a late CNS relapse; two others remain in complete remission on ongoing TT. In progressing patients, brain metastases proved to be a significant component of progression in 70%. This is a common challenge in BRAF mutant melanomas [43].

The toxicity of our sequential treatment approach seemed modest compared to data from the trials described above. In our series toxicity mostly reached Grade 1–2 intensity. Most toxicity was managed with temporary treatment interruption and steroid administration, if toxicity was typical for CKI immunologic toxicity, or withholding TT and subsequent dose reduction if toxicity was believed to be TT related (e.g., a typical non-pruritic MEK inhibitor rash). In these patients, treatment was resumed once toxicity reached grade 1 or less. In patients with recurrence of TT toxicity after re-challenge, patients were converted to alternate BRAF or MEK inhibitors (6 patients, one required a trial of 3 separate combinations to achieve acceptable levels of toxicity). A total of 21/23 patients was able to remain on therapy after toxicity resolved. Two of 23 patients had to discontinue treatment due to toxicity. These two patients required hospitalization for grade 4 biopsy-proven interstitial nephritis. In the first patient, renal impairment responded completely to treatment cessation and high dose steroids. This patient did not receive further combined therapy and continues in long-term complete response. The second patient had both interstitial nephritis, ataxia, and concomitant acute sensory-motor neuropathy. He responded dramatically to high dose steroids with the addition of IV immunoglobulin. Treatment with TT was resumed after recovery, however this patient (with a BRAF insertion) eventually died due to melanoma progression. One patient died suddenly of a sudden cardiopulmonary event.

Unfortunately, a subset (44%) of patients failed to respond to the addition of TT to PD-1 treatment. Further work will be needed to understand the mechanism(s) of resistance and to identify more active treatment options in this subset of patients. A number of these progressing patients were characterized by the presence of elevated LDH or brain metastases at progression (known adverse prognostic markers). Patients with internal BRAF rearrangements or fusions also appeared to have an extremely poor prognosis after treatment with both CKI and CKI + TT. The dichotomous response pattern in our patients is curious: Most responding patients eventually reached a complete response and virtually all initially progressing patients have died. The basis for this observation remains to be elucidated. Our clinical trial suggests that it may be possible to identify the eventual response pattern at early times (e.g., within 2–3 months) after adding TT to CKI. If verified, this potentially would allow non-responding patients to transition to clinical trials of new agents, before disease progression results in deterioration of performance status.

Our current study is intended to be hypothesis generating, with a goal of providing preliminary data to supporting development of confirmatory clinical trials. Potential limitations of this study include that it is a retrospective review of patient outcomes over a number of years involving a relatively small number of patients. Patients were treated with a number of different CKI regimens as well as TT agents based on availability over this time. It is possible that an attempt to further escalate TT agent doses might further increase responses, as may adding additional agents to overcome CKI or TT resistance mechanisms. We are hopeful that our promising results will stimulate further trials of concurrent versus sequential CKI and TT.

## Conclusions

Patients with metastatic melanoma who progress after failure of initial immunotherapy have a high mortality. In patients with BRAF mutations, continuation of PD-1 antibody treatment with cautious addition of low-dose BRAF±MEK inhibitors appears to have significant activity and may convert some patients to long term remissions. Further evaluation of this approach appears to be warranted.

## Data Availability

De-identified data supporting this manuscript is available upon reasonable request to corresponding author.

## Abbreviations

TT: targeted therapy
CKI: checkpoint inhibitor
R1: response to checkpoint inhibitors alone
R2: response to PD-1 therapy plus BRAF±MEK inhibitors
CR: complete response
PR: partial response
SD: stable disease
PD: progressive disease
LDH: lactate dehydrogenase
CNS: central nervous system
PFS: progression-free survival
OS: overall survival
TRAE: treatment related adverse event
HR: hazard ratio
BID: twice daily
QD: daily
